# Overexpression of CDCA8 Predicts Poor Prognosis and Promotes Tumor Cell Growth in Prostate Cancer

**DOI:** 10.3389/fonc.2022.784183

**Published:** 2022-04-05

**Authors:** Shun Wan, Yang He, Bin Zhang, Zhi Yang, Fang-Ming Du, Chun-Peng Zhang, Yu-Qiang Fu, Jun Mi

**Affiliations:** Department of Urology, Lanzhou University Second Hospital, Lanzhou, China

**Keywords:** prostate cancer, CDCA8, proliferation, immune infiltration, molecular mechanism

## Abstract

Human cell division cycle-related protein 8 (CDCA8) is an essential component of the vertebrate chromosomal passenger complex (CPC). CDCA8 was confirmed to play a role in promoting malignant tumor progression. However, the exact function of CDCA8 in the development and progression of prostate cancer (PCa) remains unclear. In this study, the database GSE69223 was downloaded by the gene expression omnibus (GEO) database, as well as CDCA8 expression differences in multiple tumor tissues and normal tissues were detected by The Cancer Genome Atlas (TCGA), TIMER, Oncomine, and Ualcan databases. Kaplan-Meier and Cox regression methods were used to analyze the correlation between CDCA8 expression and prognosis in PCa. We confirmed the expression of CDCA8 in PCa tissues by HPA. We also analyzed the association of CDCA8 expression with PCa clinical characteristics in the TCGA database. To further understand the role of CDCA8 in PCa, we assessed the effects of CDCA8 on PCa cell growth, proliferation, and migration *in vitro* studies. As a result, CDCA8 was significantly overexpressed in PCa cells compared with normal prostate cells. High CDCA8 expression predicts poor prognosis in PCa patients, and CDCA8 expression was higher in high-grade PCa. In addition, silencing of CDCA8 significantly inhibited PCa cell proliferation and migration. In summary, CDCA8 promoted the proliferation and migration of PCa cells.

## Introduction

Prostate cancer (PCa) is the second leading cause of cancer death in men worldwide, with nearly 1.28 million new cases and 3.59 million deaths worldwide, according to epidemiological statistics in 2018 (1). Genetic testing plays an increasingly important role in treating patients with PCa. Studies now recommend that all patients with pancreatic or metastatic PCa and those with a family history of a high incidence of Gleason high-grade PCa should undergo genetic testing. Identifying genetic mutations can guide patients to assess the risk of other cancers and identify and manage diseases in relatives (2). The human cell division cycle-associated 8 (CDCA8) protein is a chromosomal complex essential for genome transmission during cell division (3). It has been shown that the CDCA8 gene is highly expressed in breast cancer cells and that CDCA8 gene knockdown can inhibit the survival and growth of cancer cells. Another interesting phenomenon is that higher CDCA8 gene expression is positively correlated with the poor prognosis of which cancers. CDCA8 is, therefore, a key mediator of estrogen-stimulated breast cancer cell growth and survival (4). Studies have confirmed that CDCA8 plays a crucial role in mitosis, chromosome segregation, and cancer cell division (5). A study showed that CDCA8 was overexpressed in colorectal cancer. At the same time, loss of CDCA8 inhibited the growth of cancer cells and induced apoptosis (6). In addition, it has been reported that high expression of CDCA8 is significantly associated with lymph node metastasis in melanoma (7). This study aimed to evaluate the prognostic significance of CDCA8 gene expression in PCa by bioinformatics analysis of clinical features and survival information from The Cancer Genome Atlas (TCGA). We also performed *in vitro* experiments to investigate the effect of CDCA8 expression on PCa cell proliferation and invasion. Our results suggest that CDCA8 can be used to predict the prognosis of PCa patients and that high expression of CDCA8 is associated with poor prognosis in these patients.

## Materials and Methods

### Dataset and Sample Extraction

From TCGA data (https://portal.gdc.cancer.gov/), RNA sequencing (RNA-seq) data on PCa and related cancers were obtained. The data were RNA-seq data in transcripts per million reads format of TCGA and GTEx uniformly processed by the Toil process (8). Ethics committee approval was not required as the data involved in this study were obtained from the TCGA database and adhered strictly to TCGA publication guidelines.

### Screening of DEGs and Hub Genes

There were 15 PCa samples and 15 standard samples in this study. GSE69223 was downloaded from the Gene expression omnibus (GEO) database through the GEO query package to remove probes with a probe corresponding to multiple molecules. DEGs were identified by the GEO2R online analysis tool using adjusted P < 0.05 and | logFC | ≥ 1 as cut-off criteria. The GEO query package was used for data collection and download. A complex heatmap package was used to visualize the heat map. The target DEGs were imported into the Gene MANIA online database to construct a PPI network. The top 30 genes in the PPI network were selected as central genes. The cluster profile package was used for GO and KEGG enrichment analysis. The ggplot2 package was used for visualization. When the data satisfies p studies were included when p adj < 0.05 & q value < 0.2.

### Epigenetic Inheritance of CDCA8 in Urinary Tumors

We used the UCSC Xena database (http://xena.ucsc.edu/) to analyze the gene copy number, methylation, somatic mutations, gene expression of CDCA8 in urinary tumors (PCa, ACC, KIRP, and KIRC).

### Correlation Between CDCA8 and Human Immune Cells and Infiltration Level

Investigating the correlation between CDCA8 and human immune cells, the TISIDB database was used to explore CDCA8 expression in human cancer with human immune cells and chemokines. The TIMER algorithm was used to determine the possible link between CDCA8 expression and immune cell infiltration.

### Expression of CDCA8 in Pantothenic Carcinoma and Its Expression in PCa

The GSE69223 dataset downloaded from the GEO database was analyzed for CDCA8 expression using R language visualization. The expression of CDCA8 in pan-cancer was obtained using the TCGA database. Immunohistochemical pictures of CDCA8 in normal prostate and PCa were obtained from the HPA online database.

### CDCA8-Associated Signaling Pathways

To gain more insight into the function of CDCA8, GSEA was used to map the KEGG pathway and GO analysis databases. We performed GSEA using low and high CDCA8 expression datasets to identify signaling pathways differentially activated in PCa. The relevant gene pathways were selected based on the cut-off criteria FDR < 0.05 and gene size≥100.

### Cell Line and Cell Transfection

RWPE-1, a normal prostate epithelial cell line, was cultured in DMEM (G4510; Servicebio, Wuhan, China) and PCa cell lines (LNCaP, DU-145, and PC3) were cultured in RPMI-1640 (G4530; Servicebio, Wuhan, China). All cell lines were obtained from the Second Hospital of Lanzhou University and incubated at 37°C and 5% CO_2_ in a humidified incubator. All mediums were supplemented with 10% fetal bovine serum (FBS; 1276-025; Gibic, Beijing, China). Transfection was performed using Homo-CDCA8 (shRNA-CDCA8) and shRNA-negative control (shRNA-NC) lentivirus (GebePharma; Shanghai, China). LNCaP and DU-145 were cultured in six-well plates and transfected with lentivirus and Polybrene enhancer for 16 h. After 72 h, 2 μl 0.1 μg/μl puromycin was added to select stable cell lines. The shRNA single-strand sequences were as follows: Sh-CDCA8: 5’-TTGACTCAAGGGTCTTCAA-3’; Sh-NC: 5’-ACTACCGTTGTTATAGGTG-3’.

### Real-Time PCR

Total RNA was extracted from cultured cells using TRIzol (AG21101; Hunan, China) reagent following the manufacturer’s instructions and reverse transcription. Real-time quantitative PCR was performed as Yang et al. (9) described. All results for gene expression were normalized to those for β-actin. Relative quantification was performed using the 2^–ΔΔCT^ method. Primers used for qRT-PCR were as follows: CDCA8: forward primer, 5’-CCAGAGGCCTTGGGAAACAT-3’; reverse primer, 5’-AGGAACATGGCTCCTTGC-3’; GAPDH: forward primer, 5 ‘-GGAAGCTTGTCATCAATGGAAATC-3’ and reverse primer, 5 ‘-TGATGACCCTTTTGGCTCCC-3’.

### Western Blotting

Total protein samples were extracted from tissues and cultured cells. Then, samples were separated using 10% SDS-PAGE and transferred to polyvinylidene fluoride membranes. After blocking with 5% skimmed milk in Tri-buffered water for 1 h, membranes were incubated overnight at 4°C with target antibodies against the following proteins: anti-CDCA8 (1:1000, DF6115; Affinity, USA), anti-β-actin (1:2000; SA0000-1-Ig; Proteintech, Beijing, China), IRDye 800CW secondary antibody (1:20000; 926-32211; licor, Shanghai, China). β-actin was used as a reference. After three TBST washes, IRDye 800CW secondary antibody was added for 1 h at room temperature. Next, the bands were visualized by an Odyssey CLX dual-color infrared laser imaging system (CLX-0470, Genecompany, Shanghai, China). Finally, relative protein expression levels were assessed using Image J software.

### Cell Proliferation Assay

For cell proliferation analysis, selected stable transplants of sh-CDCA8 and sh-NC from LNCaP and DU-145 cells were seeded in 96-well plates (2000 cells/well). CCK-8 reagent (100 μL/well) was then added to each well and cultured at 37°C for 2 h. Cell growth at 0, 24, 48, and 72 h were analyzed with Cell Counting Kit-8 (CCK-8; Apexbio, USA). The absorbance was measured at 450 nm.

### Clonogenic Assay

Transfected LNCaP cells and DU-145 cells (shRNA-CDCA8, shRNA-NC) were seeded onto six-well plates with 700 cells per well and incubated for 14 days. They were fixed in 10% formaldehyde for 15 minutes, stained with 4% crystal violet for 30 minutes, and counted. Image J was used to calculate the number of colonies per well.

### Wound-Healing Assay

Transfected LNCaP cells and DU-145 cells (shRNA-CDCA8, shRNA-NC) were seeded into six-well plates at 400,000 cells per well and cultured in an incubator for 24 h. Take out the 6-well plate use a ruler to draw a horizontal line on the bottom of the six-hole plate with a marking pen. The distance between each horizontal line and each horizontal line is 0.5 cm. The next day, use a 200 μl tip perpendicular to the surface of the six-hole plate and draw three vertical lines from the top perpendicular to the bottom. The culture medium was sucked off, the subcultured cells were washed off with PBS, and a serum-free medium was added for culture. The last scratch widths were collected under an inverted microscope for 0 and 24 hours.

### Statistical Analysis

R (version 3.6.0), SPSS Statistics25.0 (IBM, Inc., Chicago), and ImageJ software performed all statistical analyses. To compare survival curves, we used the log-rank test to calculate HR and log-rank P values in Kaplan-Meier Plotter and GEPIA. Univariate Cox regression models were used to calculate HR and Cox P values in Progno Scan. R software and ggplot2 were used to visualize these cancers associated with CDCA8 and the poor prognosis of PCa. Differences in quantitative data between two groups were analyzed using paired or unpaired Student’s t-test, Mann-Whitney U test, or Dunnett’s test as appropriate. Two-way ANOVA with Sidak’s multiple comparisons was applied for multiple comparisons. We considered P < 0.05 as statistically significant (*P < 0.05, **P < 0.01, ***P < 0.001).

## Results

### Screening of DEGs and Hub Genes in TCGA and GEO

**Figure 1:**
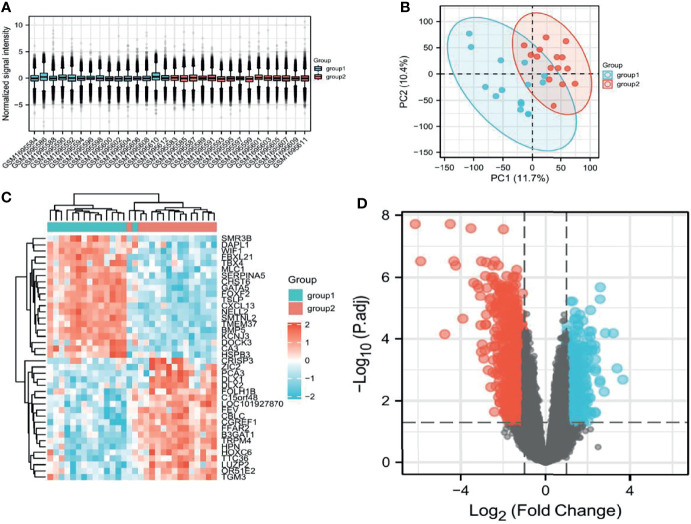
Screening of relevant PCa genes in TCGA and GEO databases. **(A)** Sample homogeneous representation in the GSE69223 dataset. **(B)**, PCA indicates that the selected samples have good heterogeneity. **(C)** Differentially expressed genes between tumors and non-tumors. **(D)** Distribution of differentially expressed genes.

After filtering the GSE69223 data set, the samples were homogeneous and normalized by box plots (**Figure 1A**). Group1 (normal) and group2 (cancer) sample groups were quite different by PCa analysis (**Figure 1B**), and **Figure 1C** shows the expression of the top 20 up-regulated and 20 down-regulated DEGs with fold change 2. A total of 1203 DEGs were detected after analysis of GSE69223, of which 380 up-regulated genes and 823 were down-regulated (**Figure 1D**). Moreover, most of these genes were up-or down-regulated in metastatic PCa. We successfully constructed the PPI network based on the degree of correlation from high to low. Finally, We selected the top 12 central genes: CDCA8, HOXC6, CRISP3, ZIC2, FEY, PTK6, NKX2-3, GCNT1, KCNC2, STX19, ALDH3B2, GPR158 (**Supplementary Figure 1**). The results indicated that CDCA8 had a significant correlation with these Hub genes. In order to better study the mechanism and immunity of a single gene, we selected CDCA8 for further study.

### Gene Methylation, Mutation, and CNV With CDCA8-Related in PCa

**Figure 2:**
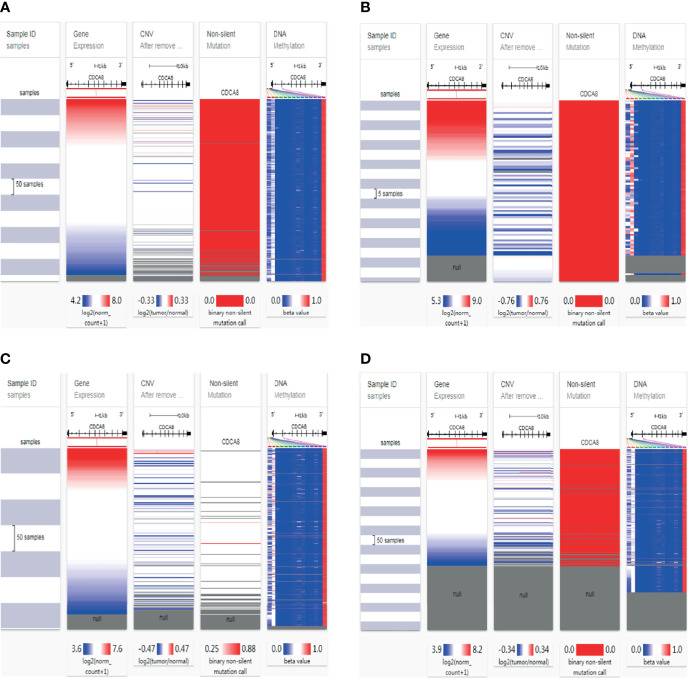
Mutation, Copy Number Variation, and Methylation Analysis of CDCA8. CNV, DNA methylation and somatic mutations in PCa **(A)**, ACC **(B)**, KIRP **(C)** and KIRC **(D)**.

To analyze the potential mutation sites and methylation modifications of CDCA8 at the genetic level in urinary tumors. We used the UCSC Xena database to analyze the epigenetic inheritance of CDCA8 in PCa, ACC, KIRP, and KIRC. The results indicated that CDCA8 expression was significantly elevated in PCa, ACC, KIRP, and KIRC. Then we assessed the cause of the elevated CDCA8 levels. The heatmap indicated that CDCA8 mRNA expression correlated with CNV and DNA methylation in PCa (**Figure 2A**), ACC (**Figure 2B**), KIRP (**Figure 2C**), and KIRC (**Figure 2D**), but not with somatic mutations in PCa, ACC, and KIRC. As a result, DNA methylation, gene mutation, and CNV are closely related to genetic and epigenetic regulation and are associated with cancer progression. Therefore, we suggest that CNV and DNA methylation may cause elevated CDCA8 levels in PCa, ACC, KIRP, and KIRC.

### CDCA8 Correlates With Immune Cell Infiltration and Chemokines in PCa

**Figure 3:**
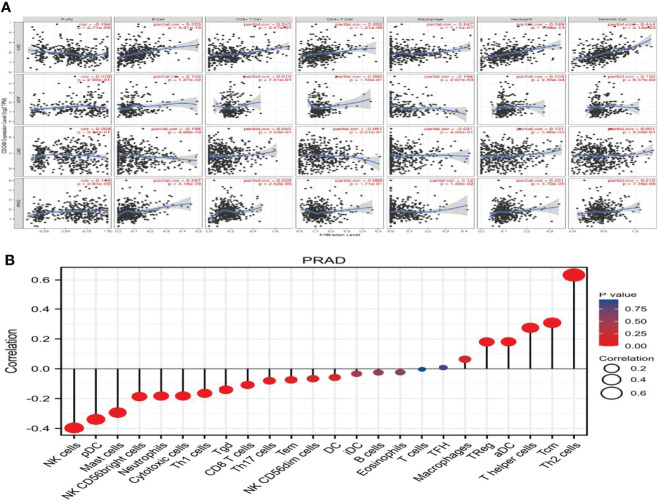
**(A)** The correlation between PCa, KIRP, KIRC, LUAD, and immune cell infiltration. **(B)** The correlation between PCa and immune factor infiltration.

We used the TISIDB online database to analyze the link between CDCA8 expression and immune cell infiltration in cancers. The results indicated that CDCA8 expression was significantly positively correlated with tumor purity, infiltration levels of B cells, CD8+T cells, CD4+T cells, and macrophages in PCa (**Figure 3**). In analyzing the association of human cancers with immune cells and chemokines, we found that CDCA8 expression was positively correlated with human immune cells Act-CD4 (rho = 0.465, p < 0.01), Th2, MeM-B and chemokines CCL in PCa (**Supplementary Figure 2**). A separate analysis of the correlation between CDCA8 and immune cell infiltration in PCa revealed that CDCA8 was positively correlated with cellular infiltration such as Th2 and Tcm.

### Expression of CDCA8 in Pantothenic Carcinoma Including PCa

**Figure 4:**
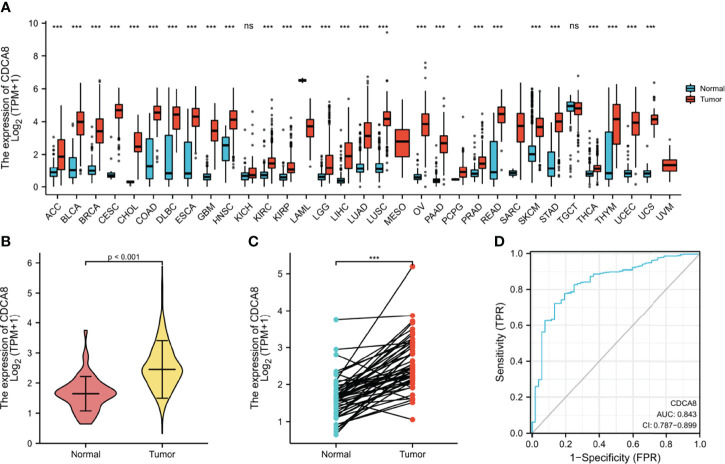
**(A)** Expression of CDCA8 in pan-cancer. **(B, C)** Differential expression analysis of CDCA8 between prostate cancer and normal tissues in TCGA database; **(D)** Sensitivity and specificity of CDCA8 in the diagnosis of PCa. ^ns^P > 0.05; *P < 0.05; **P < 0.01; ***P < 0.001.

Investigating the differential expression of CDCA8 between human tumors and normal tissues, the expression levels of CDCA8 in pan-cancer were analyzed using the TCGA database. The results showed that CDCA8 was highly expressed in ACC, BLCA, BRCA, ESCA, PRAD, and UCS cancer compared with each normal tissue (**Figure 4A**). Visual analysis showed that the expression of CDCA8 in PCa was significantly higher than that in normal tissues (**Figure 4B**), and analysis after another pairing to remove heterogeneity revealed the same results (**Figure 4C**). Receiver operating characteristic curve (ROC) was used to analyze the sensitivity and specificity of CDCA8 in the diagnosis of PCa (**Figure 4D**), and the result showed that the cut-off value of CDCA8 in the diagnosis of PCa was exact (AUC = 0.843). We then validated in GSE69223 that CDCA8 expression is significantly higher in PCa than in normal tissues (**Supplementary Figure 3B**); the above results were significantly different (P < 0.05). We obtained immunohistochemical pictures of normal prostate tissue and prostate cancer from HPA (**Supplementary Figures 3C, D**).

### High CDCA8 Expression Is Associated With Poor Prognosis in PCa

**Figure 5:**
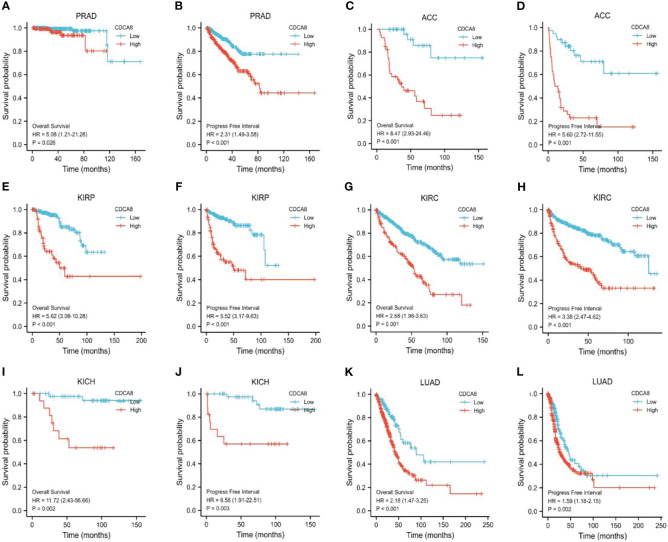
Survival analysis of CDCA8-Associated Cancers. OS and DFS in patients with PCa **(A, B)**, ACC **(C, D)**, KIRP **(E, F)**, KIRC **(G, H)**, KICH **(I, J)**, and LUAD **(K, L)**.

**Figure 6:**
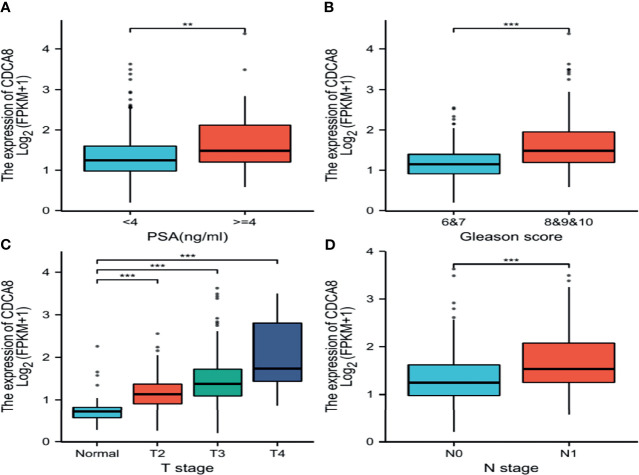
Box plot evaluating CDCA8 expression of patients with lung adenocarcinoma according to different clinical characteristics. **(A)** PSA; **(B)** Gleason score; **(C)** T classification; **(D)** N classification, (**P < 0.01, ***P < 0.001).

This study analyzed the association between CDCA8 expression with OS and DFS in PCa, ACC, KIRP, KIRC, KICH, and LUAD patients by Kaplan-Meier analysis. The results showed that high expression of CDCA8 was associated with poor OS (HR = 5.08, 95% confidence interval (CI) = 1.21-21.28, P = 0.026) and poor DFS (HR = 2.31, 95% CI = 1.49-3.58, P < 0.001) in PCa patients. Similar high CDCA8 expression was associated with worse OS and DFS in patients with ACC, KIRP, KIRC, KICH, and LUAD (**Figures 5A–L**). After Cox regression analysis by collating OS, PFI, and DSS data of human cancers associated with CDCA8, human cancer was expressed using a deep forest plot. As a result, the expression of CDCA8 is correlated with OS, PFI, and DFS in PCa (**Supplementary Figure 4**). When analyzing the correlation between CDCA8 expression and clinical characteristics of PCa patients. 499 prostate samples were downloaded from the TCGA database, including the low CDCA8 expression group (n = 249) and the high CDCA8 expression group (n = 250). The high CDCA8 expression was positively correlated with PSA > = 4 and Gleason score > 7 (**Table 1**). Further visualization findings that high CDCA8 expression might be associated with poor clinical outcomes (**Figure 6**). Univariate analysis using logistic regression indicated that high expression of CDCA8 was significantly associated with Gleason score (8&9&10 vs. 6&7, OR= 3.53, 95%CI= 2.43–5.16, p < 0.001), T classification (T3&T4 vs. T2, OR = 3.09, 95%CI= 2.12–4.54, p < 0.001), and N classification (OR = 2.73, 95%CI = 1.62–4.74, p < 0.001) (**Table 2**).

**Table 1:** Clinical features of PCa associated with CDCA8.

Characteristic	Low expression of CDCA8	High expression of CDCA8	p
n	249	250	
T stage, n (%)			<0.001
T2	124 (25.2%)	65 (13.2%)	
T3	119 (24.2%)	173 (35.2%)	
T4	3 (0.6%)	8 (1.6%)	
N stage, n (%)			0.004
N0	174 (40.8%)	173 (40.6%)	
N1	25 (5.9%)	54 (12.7%)	
M stage, n (%)			1.000
M0	221 (48.3%)	234 (51.1%)	
M1	1 (0.2%)	2 (0.4%)	
PSA(ng/ml), n (%)			0.016
<4	215 (48.6%)	200 (45.2%)	
>=4	7 (1.6%)	20 (4.5%)	
Gleason score, n (%)			<0.001
6	32 (6.4%)	14 (2.8%)	
7	145 (29.1%)	102 (20.4%)	
8	30 (6%)	34 (6.8%)	
9	41 (8.2%)	97 (19.4%)	
10	1 (0.2%)	3 (0.6%)	

**Table 2:** Univariate regression analysis associated with high CDCA8 expression.

Characteristics	Total (N)	Odds Ratio (OR)	P value
Age (>60 vs. <=60)	499	1.306 (0.917-1.862)	0.139
PSA(ng/ml) (>=4 vs. <4)	442	1.827 (0.831-4.230)	0.142
Gleason score (8&9&10 vs. 6&7)	499	3.529 (2.431-5.163)	<0.001
T stage (T3&T4 vs. T2)	492	3.092 (2.122-4.543)	<0.001
N stage (N1 vs. N0)	426	2.729 (1.619-4.742)	<0.001

The above results indicate that CDCA8 expression is significantly higher in PCa than in normal tissues. ROC curves indicate that CDCA8 can more accurately predict PCa. On the other hand, the higher the expression of CDCA8 is expressed, the worse the prognosis of PCa. Therefore, ultimately we chose the association of CDCA8 with PCa for further analysis.

### Enrichment Analysis of CDCA8 Co-Expressed Genes in PCa

**Figure 7:**
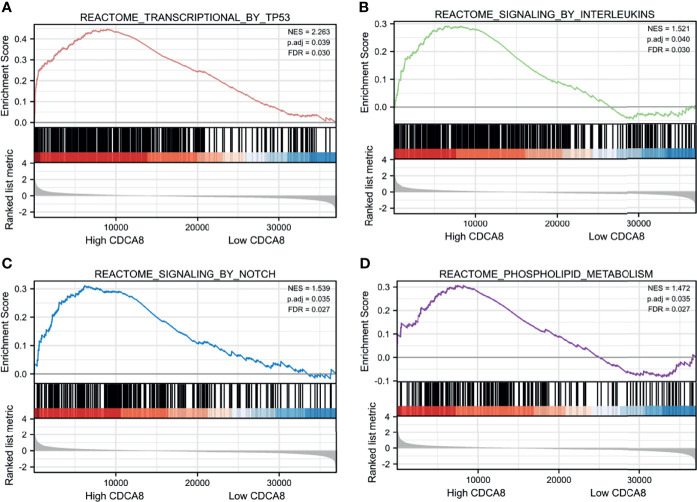
GSEA analysis of CDCA8 co-expressed genes. High CDCA8 expression showed differential enrichment in TP53 **(A)**, interleukin **(B)**, NOTCH **(C)**, and metabolic function **(D)** pathways.

To understand the signaling function of CDCA8 in PCa, GSEA was used to map KEGG pathways and GO analysis. GSEA was performed using low and high CDCA8 expression datasets to identify signaling pathways differentially activated in PCa. According to the selection criteria, the results showed four functional gene sets related to metastasis or oncogene pathways. This result indicated that high expression of CDCA8 was enriched in TP53 (ES=2.26, p=0.04, FDR=0.03), interleukins (ES=1.52, p=0.04, FDR=0.03), NOTCH (ES=1.54, p=0.04, FDR=0.03), and metabolism (ES=1.47, p=0.04, FDR=0.03) (**Figures 7A–D**). These results indicate that high CDCA8 expression shows differential enrichment in interleukin, metabolic function, TP53, NOTCH pathways.

### CDCA8 Is Significantly Expressed in PCa Cells and Silencing of CDCA8 Inhibits PCa Cell Proliferation

**Figure 8:**
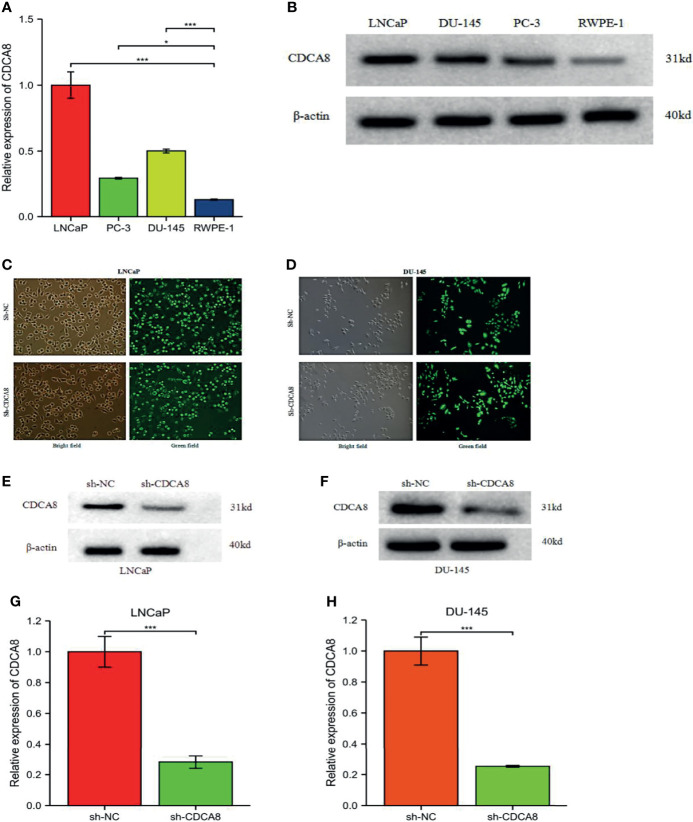
The mRNA expression levels of CDCA8 in RWPE-1 and PCa cells. Sh-NC, negative control. CDCA8 expression levels were higher in PCa cells **(A, B)**. The knockdown efficiency of LNCaP and DU-145 cells was observed by inverted fluorescence microscopy **(C, D)**. After sh-CDCA8 and sh-NC were transfected into LNCaP and DU-145 cells, the expression levels of CDCA8 were detected by qRT-PCR and Western blotting **(E-H)**, *P < 0.5; ***P < 0.001; two-tailed t-test.

We performed cell experiments to explore whether CDCA8 promotes PCa progression *in vitro* experiments. We first examined CDCA8 expression in prostate normal epithelial cells RWPE-1 versus PCa cell lines (LNCaP, DU-145, and PC-3). The results showed that CDCA8 was overexpressed in LNCaP, DU-145, and PC-3 cells compared with RWPE-1 (**Figures 8A, B**). For the following experiments, we selected LNCaP cells with the highest CDCA8 expression and DU-145 cells for subsequent experiments.

**Figure 9:**
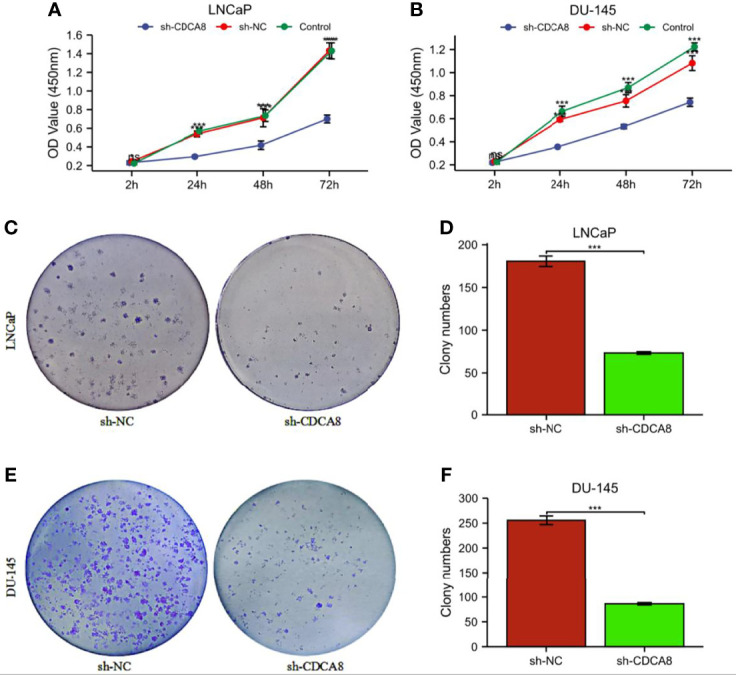
The proliferation ability of PCa cells was significantly attenuated after the knockdown of CDCA8. **(A, B)** Cellcountingkit-8 (CCK8) analysis showed that down-regulation of CDCA8 inhibited the growth of LNCaP and DU-145 cells (^ns^P > 0.05; *P < 0.05; ***P < 0.001. two-way ANOVA). **(C-F)** In a colony formation assay, silencing of CDCA8 reduced the number of colonies in LNCaP and DU-145 cells (***P < 0.001 versus sh-NC; two-tailed t-test). NC, negative control.

In order to investigate the effect of CDCA8 on the growth of PCa cells. A CCK-8 assay was performed to detect cell proliferation using stable transfer LNCaP and DU-145 cells obtained by sh-CDCA8 and sh-NC knockdown. LNCaP and DU-145 cells were transfected using shRNA-CDCA8 and shRNA-NC. The transfection efficiency was observed at 80% using an inverted fluorescence microscope after 72 h (**Figures 8C, D**). The knockdown efficiency was further verified using RT-PCR and Western blot experiments (**Figures 8E–H**). Our results indicated that down-regulation of CDCA8 significantly inhibited the proliferation of LNCaP and DU-145 cells (**Figures 9A, B**). In the colony formation assay, knockdown of CDCA8 also significantly reduced the number of cell colonies compared with sh-NC-transfected cells (**Figures 9C–F**). Both CCK8 cell proliferation and colony formation assays indicated that the decrease in CDCA8 expression inhibited the proliferation of PCa cells.

### CDCA8 Silencing Reduces the Migratory Capacity of PCa Cells

**Figure 10:**
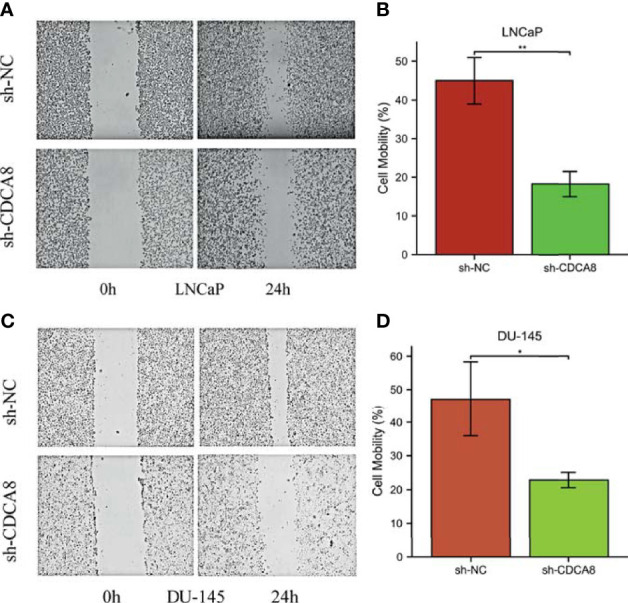
Knockdown of CDCA8 inhibited the migration of PCa cells in vitro. **(A, C)** Wound healing assay, LNCaP and DU-145 at 0 and 24 h, respectively. **(B, D)** Relative migration distance was analyzed in LnCap and DU-145 cells, respectively. *P < 0.05; **P < 0.01; two-tailedt-test.

We performed a wound-healing assay to investigate whether CDCA8 silencing affects PCa cell migration. The results showed that in LNCaP and DU-145 cells, wound healing was inhibited in sh-CDCA8-transfected cells compared with sh-NC-transfected cells for LNCaP (**Figures 10A, B**), DU-145 (**Figures 10C, D**) at 0 h and 24 h. The relative migration distances were analyzed in LNCaP and DU-145 cells, respectively. The results indicated that the cell migration rate of the sh-CDCA8 phase was lower than that of the sh-NC group, and the difference between the two groups was statistically significant (P < 0.01). These results indicate that CDCA8 promotes the migration of PCa cells and that the knockdown of CDCA8 inhibits their migration.

## Discussion

As stated in an epidemiological survey study that the incidence of PCa increased by 169.11% in 2019 compared with 1990 (10). Prostate specific antigen (PSA) is of great value in diagnosing of PCa. It is an FDA-approved biomarker for PCa (11). However, unnecessary biopsies and overtreatment in clinical practice are rising due to the low specificity of PSA in diagnosis (12). After the normal epithelium of the prostate developed into a tumor, the expression of PSA increased significantly. In people over 60 years of age, PSA production also increases, thereby reducing the sensitivity of PSA detection (13). Also, there was no significant correlation between PSA levels and PCa severity (14). Current target therapy is challenging in diagnosing and treating PCa (15). Therefore, exploring new PCa diagnostic and therapeutic targets has important clinical significance. Recent studies of high-throughput gene chips for analysis of patient average and tumor tissue samples have provided us with an opportunity to discover and explore the entire molecular landscape of tumors at various levels, from copy number changes and somatic mutations at the genomic level to altered gene expression at the transcriptional level (16–18). Microarrays currently have very few clinical applications. This limitation is broken when the advent of detecting many genes by gene profiling. However, there is still a lack of independent reliability, reproducibility, and complex statistical analysis for clinical application. At the same time, experimental identification of critical genes on a genome-wide scale is very time-consuming and laborious. An optimal method that can be processed by routine analysis needs to be developed to put these expression profiles into clinical practice as soon as possible. In addition, there is a clear need to improve our ability to enhance PCa patients at high risk of metastasis and recurrence after radical prostatectomy for PCa. The challenge of accurately predicting PCa metastasis and recurrence may be partially attributed to the complex pathways that promote disease development (19). CDCA8 plays a vital role in various tumor-related processes. In pancreatic ductal adenocarcinoma, CDCA8 promotes tumor cell proliferation (20). Consumption of CDCA8 leads to cell cycle arrest in the G2/M phase, increased DNA damage and apoptosis, and enhanced sensitivity of ovarian cancer cells to cisplatin and olaparib (21). Through the ROCK signaling pathway, CDCA8 knockdown may inhibit cancer cell proliferation and invasion (22). However, the importance of CDCA8 in PCa has not been fully elucidated. In a bioinformatics article, Songz et al. (23) built a PPI network through the KEGG database to analyze the interactions of visualized central genes. The discovery of CENPA, KIF20A, and CDCA8 may promote the development and progression of PCa, which may be new therapeutic targets and biomarkers for PCa diagnosis and prognosis. Some essential genes (including CDCA8) were validated and found to be associated with tumor stage, metastasis, biochemical recurrence, and survival. They suggested that these central genes can control cellular processes and have high clinical value. This study is the first to report that they are upregulated in PCa and correlate with survival in PCa patients. However, the authors only stated that high CDCA8 expression was associated with poor prognosis in PCa patients in this study. Nevertheless, not studied the epigenetic, genetic mutation role of CDCA8 in PCa patients and *in vitro* studies. Therefore, in our study, we further investigated the epigenetic, genetic mutation role of CDCA8 in PCa patients and further validated the role of CDCA8 in PCa patients with *in vitro* experiments. Although some prominent pathways involving AR, SPOP, MYC, RB1, and PTEN-related pathways also play a crucial role in PCa. In these complex and comprehensive processes, these central genes are involved in almost all critical cellular pathways, given that they can interact with many proteins (24–27). In another study, the authors analyzed a total of 367 PCa cases through the Cancer Genome Atlas database and performed weighted gene co-expression network analysis. The selected four central genes were CKAP2L, CDCA8, ERCC6L, and ARPC1A. The results indicate that these four central genes can distinguish tumor from normal tissue and are promising biomarkers for lymph node metastasis of PCa (28). This study provides novel insights that explain the mechanism of lymph node metastasis of PCa at the molecular level. The identified central genes may become potential biomarkers and therapeutic targets for precise diagnosis and treatment in the future. The above studies provide a more detailed molecular mechanism for lymph node metastasis with biochemical recurrence in PCa patients and provide clues for potential biomarkers and therapeutic targets. To get a reliable conclusion, we first explored CDCA8-promoted PCa progression in the TCGA and GSE69223 databases and then validated this conclusion in our experiments. In 15 pairs of PCa and prostate normal tissues from the TCGA database, CDCA8 expression was significantly higher in cancer than in normal controls. Similarly, in cell experiments, the content of CDCA8 was significantly higher in PCa cell lines than in normal prostate cells. High CDCA8 expression was associated with advanced N stage and lymphovascular invasion. In addition, CDCA8 expression gradually increased from stage I to stage III in the TCGA and GSE69223 databases. Therefore, we hypothesize that CDCA8 may be a cancer-promoting biomarker for PCa. We first validated our hypothesis in a public database. The high expression of CDCA8 in the TCGA database showed poor OS, DSS, and PFI, which confirmed the poor prognosis of the high expression of CDCA8 in PCa. All these findings indicate that CDCA8 plays a vital role in the carcinogenesis and progression of PCa. It has been stated that CDCA8 is highly expressed in LUAD cells, while miR-133b is lowly expressed, and that the promoting effect of CDCA8 on LUAD cell proliferation, migration, and invasion can be regulated by miR-133b (29). Future studies could further investigate how CDCA8 promotes prostate cancer proliferation and metastasis. Genetic testing has an extensive range of applications. Over the past few decades, this technology has made significant progress, and genetic defects in humans can be confirmed by genetic testing (30). Moreover, genetic testing can determine the appropriate targeted drug for cancer patients, which significantly improve the survival rate of cancer patients and avoid overtreatment to achieve precise treatment, thereby improving the quality of life and improving the prognosis (31, 32). It is not a problem to obtain 99% accurate DNA sequences based on the current technology. The main problem is what the ATCG combination in these DNA sequences represents and how much association is with human health remains to be solved. Monogenic diseases are relatively simple, and the corresponding relationship is also relatively easy to study. However, monogenic diseases are rare, so people often pay attention to highly prevalent complex diseases. Such as tumors, diabetes, and cardiovascular diseases are usually many opportunities to interact and participate in environmental factors (33, 34). Therefore, the limitations of genetic testing applications in risk inference for complex diseases are more pronounced. Moreover, the basic research of the CDCA8 gene in PCa needs further study. Alternatively, our study lacks clinical samples from our hospital, and future work will be needed to investigate the effects of CDCA8 *in vivo*.

## Conclusion

In conclusion, our findings suggest that CDCA8 is significantly upregulated in PCa cell lines. High expression of CDCA8 correlates with tumor histological grade of PCa and predicts poor prognosis. Knockdown of CDCA8 inhibits PCa cell proliferation. Therefore, CDCA8 can serve as a promising diagnostic and prognostic biomarker and a new therapeutic target in PCa.

## Data Availability Statement

The original contributions presented in the study are included in the article/**Supplementary Material**. Further inquiries can be directed to the corresponding author.

## Author Contributions

SW wrote and edited the manuscript. YH edited the manuscript. BZ performed the statistical analyses and generated the figures. ZY, F-MD, C-PZ, and Y-QF collected the public data. JM revised and reviewed the manuscript. All authors contributed to the article and approved the submitted version.

## Conflict of Interest

The authors declare that the research was conducted in the absence of any commercial or financial relationships that could be construed as a potential conflict of interest.

## Publisher’s Note

All claims expressed in this article are solely those of the authors and do not necessarily represent those of their affiliated organizations, or those of the publisher, the editors and the reviewers. Any product that may be evaluated in this article, or claim that may be made by its manufacturer, is not guaranteed or endorsed by the publisher.
