# Supplementary material for: Overexpression of CDCA8 Predicts Poor Prognosis and Promotes Tumor Cell Growth in Prostate Cancer
Source: Front Oncol. 2022 Apr 5;12:784183. doi: 10.3389/fonc.2022.784183 (PMC9016845; doi:10.3389/fonc.2022.784183)
Supplement: Supplementary file 1 [file DataSheet_1.zip › Supplementary Figure 2.docx]

**CDCA8 correlates with immune cells and chemokines in cancers**

**
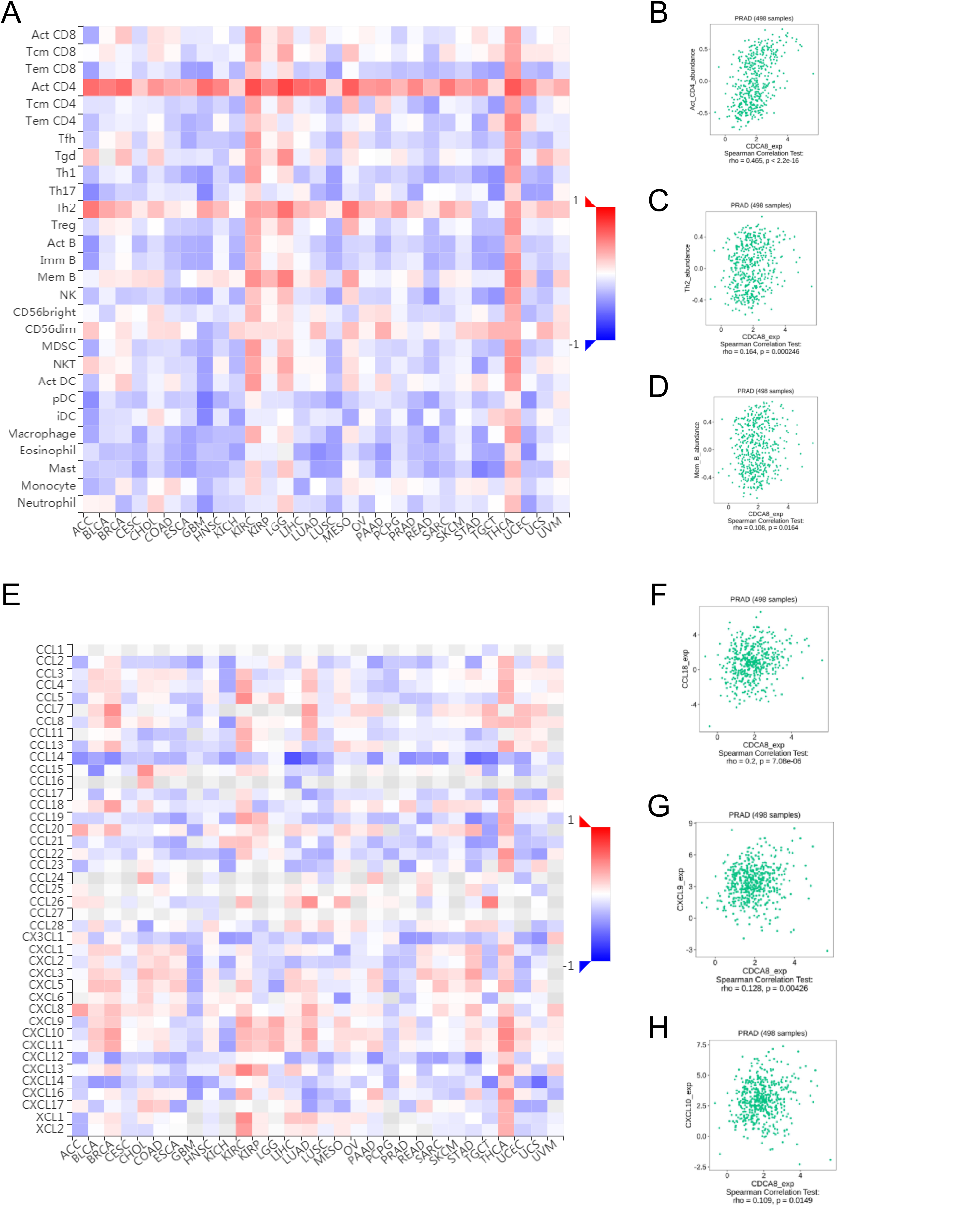
**

Figure S2. Correlation between CDCA8 and human immune cells and chemokines. ( A) CDCA8 expression in human cancer is distributed in human immune cells. Correlation analysis between Act-CD4, Th2, MeM-B, and CDCA8 in PCa (B-D); (E) Correlation analysis between CDCA8 expression and chemokines in human cancer. CCL18, CXCL19, CXCL10 were positively correlated with CDCA8 expression in PCa (F-H).
