# Supplementary material for: Overexpression of CDCA8 Predicts Poor Prognosis and Promotes Tumor Cell Growth in Prostate Cancer
Source: Front Oncol. 2022 Apr 5;12:784183. doi: 10.3389/fonc.2022.784183 (PMC9016845; doi:10.3389/fonc.2022.784183)
Supplement: Supplementary file 1 [file DataSheet_1.zip › Supplementary Figure 3.docx]

**Differential expression of CDCA8 in PCa versus normal prostate tissues**

**
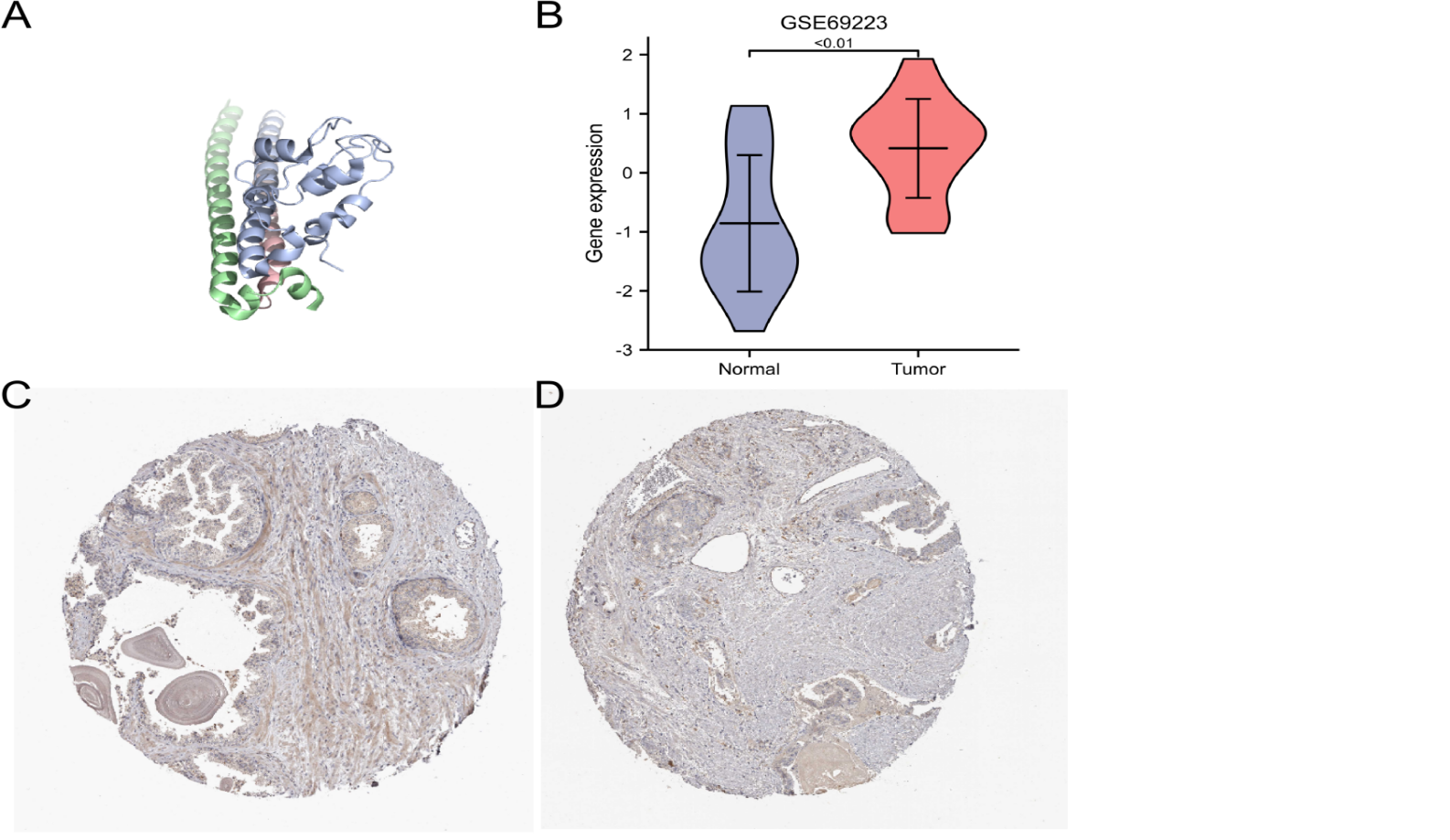
**

Figure S3. Expression of CDCA8 in PCa and normal prostate tissues. (A) Biological structural formula of CDCA8 molecule. (B), the differential expression of CDCA8 in PCa versus normal. (C) CDCA8 expression in normal prostate tissue. (D) CDCA8 expression in PCa.
