# Supplementary material for: Overexpression of CDCA8 Predicts Poor Prognosis and Promotes Tumor Cell Growth in Prostate Cancer
Source: Front Oncol. 2022 Apr 5;12:784183. doi: 10.3389/fonc.2022.784183 (PMC9016845; doi:10.3389/fonc.2022.784183)
Supplement: Supplementary file 1 [file DataSheet_1.zip › Supplementary Figure 4.docx]

**CDCA8 overexpression indicates a poor prognosis**

**
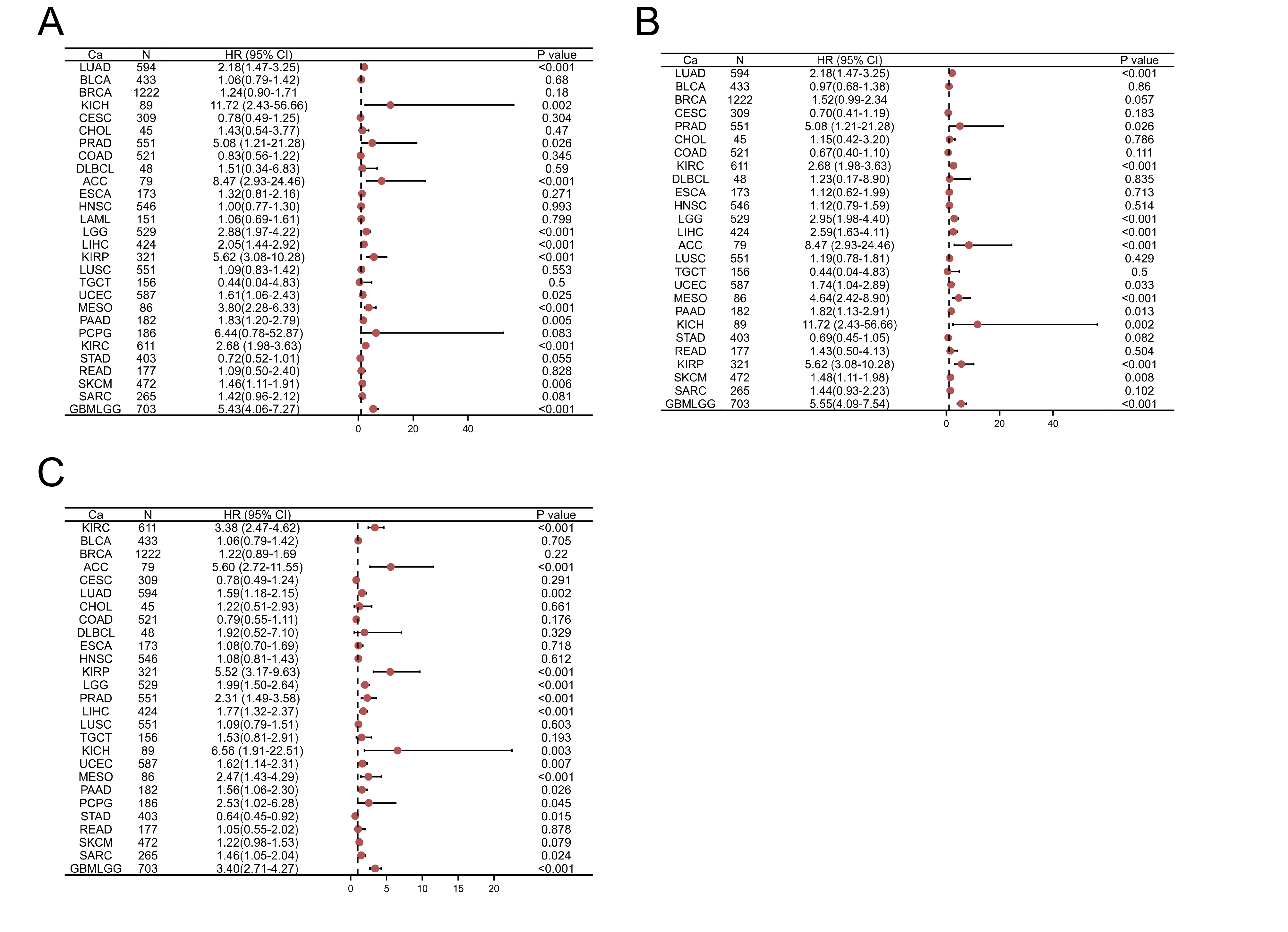
**

Figure S4. Forest map of Cox regression analysis of OS (A), PFI (B), DSS (C) in human cancers.
