# Supplementary material for: Overexpression of CDCA8 Predicts Poor Prognosis and Promotes Tumor Cell Growth in Prostate Cancer
Source: Front Oncol. 2022 Apr 5;12:784183. doi: 10.3389/fonc.2022.784183 (PMC9016845; doi:10.3389/fonc.2022.784183)
Supplement: Supplementary file 1 [file DataSheet_1.zip › Supplementary Figure 1.docx]

**Construction of PPI Network and Gene Function Analysis**


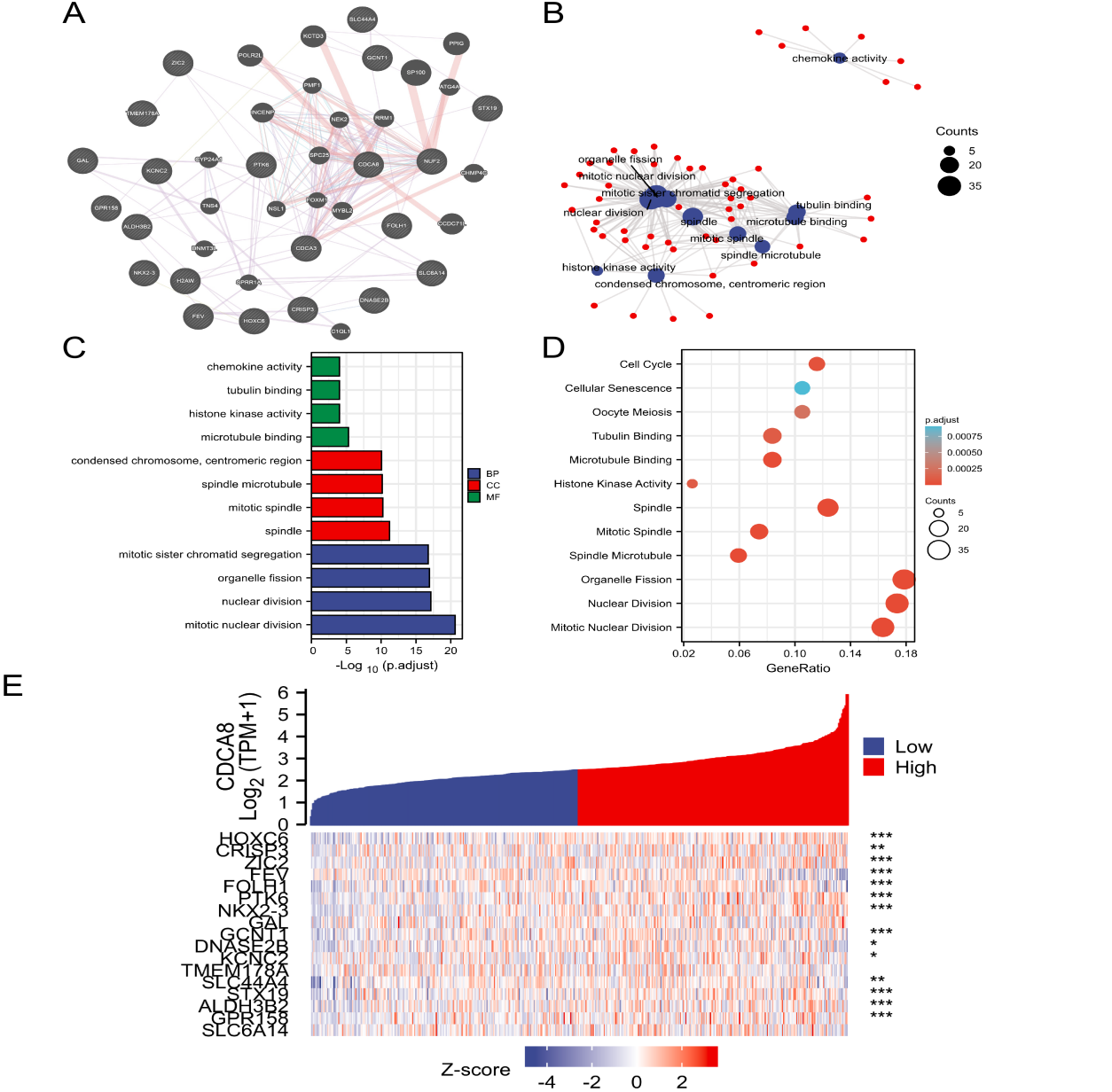


Figure S1. Gene network diagram, GO and KEGG analysis related to PCa. (A-D) Select the biological function of the gene in the sample and the biological reaction involved. (E) Correlation analysis of genes associated with CDCA8.
